# Use of a plant-based flavonoid blend in diet for growth, nutrient digestibility, gut microbiota, blood metabolites, and meat quality in broilers

**DOI:** 10.5455/javar.2024.k848

**Published:** 2024-12-27

**Authors:** Shathi Akter, Md. Aliar Rahman, Mahbubul Pratik Siddique, Md. Abul Hashem, Rakhi Chowdhury

**Affiliations:** 1 Department of Animal Nutrition, Faculty of Animal Science and Veterinary Medicine, Habiganj Agricultural University, Habiganj, Bangladesh; 2 Department of Animal Nutrition, Faculty of Animal Husbandry, Bangladesh Agricultural University, Mymensingh, Bangladesh; 3 Department of Microbiology and Hygiene, Faculty of Veterinary Science, Bangladesh Agricultural University, Mymensingh, Bangladesh; 4 Department of Animal Science, Faculty of Animal Husbandry, Bangladesh Agricultural University, Mymensingh, Bangladesh

**Keywords:** Broilers, Flavonoid blend, Gut health, Digestibility, Performance

## Abstract

**Objectives:**

This study aimed to determine the optimal doses of a flavonoid blend (FB) for enhancing cost-efficient production, digestibility, gut-beneficial microbiota, serum metabolites, and meat quality in broilers.

**Materials and Methods:**

For 35 days, 280-day-old chicks (Cobb-500) were randomly allocated to four groups, each containing 70 birds, with 5 replicates. Birds were given FB (gm/kg) at the levels of 0.0, 0.2, 0.4, and 0.6 in a basal diet (corn-soya-based) and designated as the control, 0.2 FB, 0.4 FB, and 0.6 FB groups, respectively. At 35 days, 15 birds from each group were slaughtered to analyze cecum microbiota, serum profiles, meat, and bone quality.

**Results:**

Compared with the control, birds given FB linearly showed better feed intake and overall performance, with the optimum results observed in 0.6 FB. Birds fed FB resulted in linear, quadratic, and cubic improvements in digestibility, with the 0.6 FB group presenting 12% more (*p* < 0.01) crude protein digestibility than the control. Birds offered either 0.4 FB or 0.6 FB increased (*p* < 0.01) the population of beneficial bacteria while reducing (*p* < 0.01) pathogenic bacteria in the cecum compared to the control. Birds fed 0.6 FB showed substantial improvements in beneficial serum metabolites and liver health, along with reduced bad cholesterol compared with the control. Although FB was unaffected (*p* > 0.05) by dressed yield, meat composition, lightness, or bone characteristics, the 0.6 FB group showed substantially (*p* < 0.01) more meat redness and bone ash percentage in broilers than in the control.

**Conclusions:**

Supplementing 0.6 gm FB/kg of diet improved growth performance, enhanced digestibility, increased beneficial gut microbiota and serum metabolites, and ameliorated meat quality in broilers.

## Introduction

The demand for safe and healthy broiler meat is swiftly rising globally. It is estimated that by 2030, broiler meat will account for almost 41% of the global meat protein supply [1]. This rapidly growing demand is attributed to the fact that broiler meat is economical, nutritious, easily palatable, and digestible, as well as widely preferred by people of all ages and religions [2]. However, the increasing restrictions on the use of antibiotic growth promoters and the development of resistance to some commonly used antimicrobials have led to a greater incidence of dysbiosis in broilers [3]. Dysbiosis is an imbalance in the gut microbiota that is closely associated with the overall health and disease status of the host [4]. Besides, heat stress, a common challenge in subtropical regions, can further reduce beneficial microbes and increase pathogenic microbial load in the gut [5]. It leads to the production of toxins in the gut, which can disrupt mucus secretion and composition and cause focal ulcerations of the mucosa, ultimately reducing nutrient digestibility and absorption [6]. Additionally, it results in decreased production of bacteriostatic peptides in the pancreas, reduced immunoglobulin secretion, and a rush chronic inflammatory response [6]. These conditions contribute to significant economic losses, including lower growth rates, worse feed conversion ratio (FCR), and poor meat quality in broilers. To address symbiosis issues, phytomolecules, particularly flavonoids from plants, have shown effectiveness in reducing pathogenic microbes while enhancing beneficial microbes in the gut. This improvement leads to better immunity, nutrient utilization, growth performance, and meat quality in broilers [7].

A plant-based guaranteed flavonoid blend (FB), namely, Anta^®^ Phyt Optidose, derived from hops, liquorice, and Arabic gum, is a growth-promoting feed additive with anti-inflammatory properties. Moreover, it has the properties of reducing gram-positive bacteria, thus enhancing the immunity of broilers. Its main component is hops (*Humulus lupulus* L.), which possess a broad range of phytomolecules, particularly flavonoids such as rutin, quercetin, luteolin, and isoxanthohumol [8]. Based on the previous findings [9,10], rutin, though considered an expensive additive, is effective at a cost-efficient dose of 0.5 gm/kg of diet in broilers. At this dose, rutin shows improved growth performance, better gut health, strong immunity, and elevated serum antioxidant status in broilers. Besides, quercetin is highly effective in scavenging free radicals, which promotes cell growth and differentiation, as well as supplementation of 0.25–1.0-gm quercetin per kg diet has been shown to improve growth performance, enhance intestinal morphometry, and create a healthier gut environment by increasing *Lactobacillus* populations and reducing coliform counts in broilers [11]. In addition, liquorice extracts at lower levels are effective in preventing lipid peroxidation and protecting against free radical formation, contributing to better growth, carcass yield, and gut health in broilers [12,13]. Furthermore, Arabic gum, when included in broiler diets at low levels between 0.25% and 0.75%, positively affects feed intake (FI), overall performance, nutrient digestibility, microbiota balance, and gut health [14].

According to all of these findings, individual supplementation of either rutin, quercetin, liquorice, or Arabic gum extracts shows improved broiler health as well as meat quality by enhancing antioxidant status, reducing free radical formation, and boosting immunity in broilers. Though there are some reports on the use of particular flavonoids’ effects on the growth performance, immunity, and antioxidant capacity of broiler chickens [15-17], to our knowledge, no studies have yet investigated the use of plant-based FBs/mixtures in broiler diets as a potential feed additive to enhance gut microbiota composition, nutrient digestibility, growth performance, serum metabolite profiles, as well as meat and bone quality. In addition, synthetic flavonoids are expensive, and using them in higher doses in broiler production improves health status but is not cost-effective [9]. Furthermore, with the increasing concern about the effective use of FB as feed additives in broiler production, it is essential to determine a cost-effective dose. Therefore, this research was designed to identify an optimal dose of plant-based FB to ensure optimum performance, better meat quality, and health status of broilers.

## Materials and Methods

### Ethical approval

The procedures of this research were approved by the Ethical Standards of the Research Committee at the Bangladesh Agricultural University Research System, Bangladesh (1414/BAURES/ESRC/AH/72).

### Birds and diets

A total of 280-day-old mixed broiler chicks (Cobb-500) were procured from a local commercial hatchery. For 35 days, the birds were randomly assigned to four dietary groups (five replicates per group and 14 birds per replication) in a completely randomized design and fed basal diets supplemented with 0.0-, 0.2-, 0.4-, and 0.6-gm FB per kg diet and taken as 0 FB (control group), 0.2 FB, 0.4 FB, and 0.6 FB, respectively. The basal diet contained mainly corn and soybean meal and was formulated to meet the nutrient requirements of Cobb-500 broilers [18]. The corresponding ingredients and nutritional composition are listed in Table 1. Commercially available FB named Anta^®^ Phyt Optidose was purchased from Kazi Agro Ltd., Bangladesh. This purchased FB (100%) was prepared from the extracts of herbs named hops, liquorice, and Arabic gum. Birds were reared in wire cages and given a starter diet from days 1 to 14 [21.61% crude protein (CP) and 2982-kcal metabolizable energy (ME)] and a grower diet from days 15 to 35 (20.65% CP and 3051-kcal/kg ME).

### Management and sample collection

All the cages were fitted with feeders and drinkers, which were cleaned regularly and filled with diets and clean water, respectively. Birds had unlimited access to feed (mash form) and fresh, clean drinking water. The lighting program and vaccination schedule were maintained in accordance with Cobb-500 commercial broiler management guidelines, and biosecurity was maintained strictly over the experimental period. The birds were weighed as soon as they were received, and their weight was then recorded every week. FI and mortality (if any) data were recorded daily. The FCR was calculated at the end of the trial as the ratio of FI to weight gain (gm feed/gm gain). For the determination of nutrient digestibility, a chromium oxide (Cr_2_O_3_) marker was added at the rate of 0.50% in the diets and supplied from days 29 to 35. Excreta were collected from 32 to 34 days of age and stored at −20°C in a freezer. Later, frozen excreta samples were thawed, homogenized, dried, ground, and stored for further analysis. At the end of the experiment, birds were slaughtered, and blood, meat, and bone samples were collected.

**Table 1.** Ingredients and nutritional composition of experimental diet.

Ingredients	Amount (%)	
	Starter (1–14 days)	Grower (15–35 days)
Corn	49.00	51.80
Rice polish	4.50	4.00
Soybean meal	38.50	36.00
Soybean oil	4.00	4.50
Limestone	1.40	1.55
Di-calcium-phosphate	1.80	1.35
Methionine	0.10	0.10
Lysine	0.10	0.10
Broiler premix^1^	0.30	0.30
Common salt	0.30	0.30
Nutrient composition (%, as fed basis)		
Crude protein	21.61	20.65
Crude fiber	5.07	5.00
Lysine^2^	1.39	1.30
Methionine^2^	0.46	0.44
Calcium^2^	0.96	0.92
Available phosphorus^2^	0.45	0.36
Metabolizable energy (kcal/kg)^2^	2982	3051

^1^Each kg broiler premix contained: 13.50 IU vitamin A, 1.50 IU vitamin D3, 50 mg a-DL-tocopherolacetate, 2-mg menadione, 3-mg thiamine, 5-mg riboflavin, 4-mg pyridoxin, 15-pg cobalamine, 200-pg folsaure, 60-pg nicotenic acid, 30-mg calcium pantothenate, 750-mg choline, 150-mg ascorbic acid. ^2^Calculated value.

Just after collection, blood samples were centrifuged at 3410 revolutions per minute for 15 min, and separated serum samples were collected into an Eppendorf tubes and then stored at -20°C until further analysis [19]. Immediately after slaughtering the birds, the right portion of breast samples was collected, cleaned with water, rubbed with a soft cloth, and kept in the refrigerator at 4°C for 24 h. Then, the samples were taken out of the freezer and waited until room temperature. The CR-410 colorimeter (Minolta, Japan) was placed on the cranial surface of each sample to measure and record the lightness (L*), redness (a*), and yellowness (b*) values. Subsequently, the saturation index (SI) was calculated [19]. The breast meat samples were first minced. Then, the minced meat and cecal digesta samples were each separately added to distilled water and stirred. The pH of both samples was measured individually using a Hanna pH meter (PBS-25, China).

In addition, the cecal digesta were aseptically gathered in marked Eppendorf tubes and stored at 4°C. Then, the cecal digesta were processed for bacterial count. Approximately 1 gm of cecal digesta was diluted using the 10-fold serial dilution method with sterile phosphate buffer solution, and the 10⁻⁵ dilution was used for drop plating on agar media. Plate count, eosin methylene blue, salmonella–shigella, mannitol salt, and De Man Rogosa and Sharpe agar (HiMedia, Mumbai, India) were used to determine the total viable, total *Escherichia coli*, total *Salmonella*, total Staph­ylococcal, and total probiotic bacterial count, particularly *Lactobacillus* spp., respectively [20]. The incubation period was 24 h at 37°C, except for *Lactobacillus* spp., which was incubated for 48 h. The colonies for each bacterial population were manually counted, and the bacterial counts were expressed as log₁₀ colony-forming units per gm of cecal content. Shank bones were collected, measured in length and width, and then measured for dry weight after drying at 100°C for 24 h and then ignited at 600°C for 24 h.

### Calculation and chemical analysis

Crude protein conversion ratio (CPCR), metabolizable energy conversion ratio (MECR), and cost analysis were calculated as mentioned by Chowdhury et al. [21]. The economic implications of adding FB were evaluated using the benefit-to-cost ratio (BCR) according to Chowdhury et al. [21]. The total cost was calculated by considering the market prices of the chick, test material, feed ingredients, and overall management costs. The return, however, is the selling price of the live birds. For BCR, the profit is divided by the total cost. When the BCR value is greater than 1, it means that the benefits of production outweigh the production costs; when it is less than 1, it means the opposite. Samples of diets and excreta were analyzed for proximate composition following the standard methods [22], and nutrient digestibility was determined according to Chowdhury and Koh [23]. The biochemical attributes of serum, including a lipid profile, protein indices, liver enzymes’ activity, and serum minerals, particularly calcium (Ca) and phosphorus (P), were analyzed through the Urit-810 Bio-Analyzer using different assay kits following the manufacturer’s instructions. The serum lipid profile was assessed by measuring triglycerides, total cholesterol, and high-density lipoprotein cholesterol (HDL-C). In contrast, low-density lipoprotein cholesterol (LDL-C) and very low-density lipoprotein cholesterol (VLDL-C) levels were calculated following the formulas described by Redoy et al. [18]. Furthermore, serum total protein, albumin, and uric acid were quantified, while serum globulin was calculated as per the methodology of Rahman et al. [24]. Additionally, the activities of serum liver enzymes, including aspartate aminotransferase (AST), alanine aminotransferase (ALT), and alkaline phosphatase (ALP), were determined according to Rahman et al. [24].

### Statistical analysis

The datasets from the current experiment were subjected to statistical analysis using IBM SPSS software (Version 23.0, IBM Corp., USA) via one-way ANOVA. Duncan’s multiple range test was employed to identify significant differences among groups, while orthogonal polynomial contrasts were used to evaluate linear, quadratic, and cubic effects of different doses of FB. The results are reported as mean values with standard deviations, and statistical significance was taken at *p* < 0.05.

## Results

### Performance traits

FB at various doses (0.0-, 0.2-, 0.4-, and 0.6-gm/kg diet) linearly increased final body weight, body weight gain, and average daily gain of broilers (*p* < 0.05, Table 2). The group receiving a 0.6-gm FB/kg diet demonstrated around 9.50% higher average daily gain and body weight gain compared with all other groups (*p *< 0.05). Compared with the control, FI was linearly improved (*p* = 0.02), and the best FCR was obtained in the 0.6 FB group. Additionally, FB supplementation exhibited a linear tendency to improve both the CPCR and the MECR.

### Apparent nutrient digestibility and cecal microbial population

Supplementing FB to birds led to linear, quadratic, and cubic improvements in apparent nutrient digestibility, with the 0.6 FB group showing approximately 12% greater CP digestibility compared with the control group (*p *< 0.01, Table 3). This FB supplementation, by lowering pH levels, also significantly decreased (*p* < 0.01) the counts of pathogenic bacteria such as coliforms, *Salmonella*, and *Staphylococcus. *. Furthermore, it significantly increased (*p *< 0.01) the population of probiotic bacteria, particularly* Lactobacillus* spp., with the 0.4 FB and 0.6 FB groups showing pronounced effects.

### Serum biochemical indices

The data presented in Table 4 demonstrate that FB supplementation had both linear and quadratic effects (*p *< 0.05) on serum lipid profiles. Specifically, compared with the control group, FB supplementation significantly decreased serum triglycerides, cholesterol, and LDL-C levels in the 0.6 FB and 0.4 FB groups (*p *< 0.05). Moreover, serum HDL-C levels increased by 28% in the 0.6 FB group compared with the control (*p *< 0.05). FB supplementation did not affect serum total protein, globulin, or uric acid levels (*p* > 0.05); however, it linearly increased serum albumin concentrations (*p* < 0.05). A dose-dependent reduction in liver enzymes, including ALT, AST, and ALP, was observed, with ALT and AST levels decreasing by approximately 62% and 6%, respectively, in the 0.6 FB group compared with the control. Additionally, FB supplementation had no significant effects on serum Ca or P concentrations (*p* > 0.05).

### Meat quality and bone characteristics

Although dressed yield, meat dry matter, CP, and ether extract content were not affected, FB supplementation significantly influenced breast meat color (*p* > 0.05, Table 5). The L* value of breast meat remained consistent across all groups (*p *> 0.05), whereas both a* and b* values exhibited significant linear improvements (*p* < 0.05). Among all FB-supplemented groups, the 0.6 FB group remarkably improved breast meat a* and SI value (*p *< 0.01). While bone parameters showed no significant changes, ash content increased linearly, with the 0.4 FB and 0.6 FB groups demonstrating 12%–15% more ash content than the control group.

### Cost analysis

**Figure 1.**
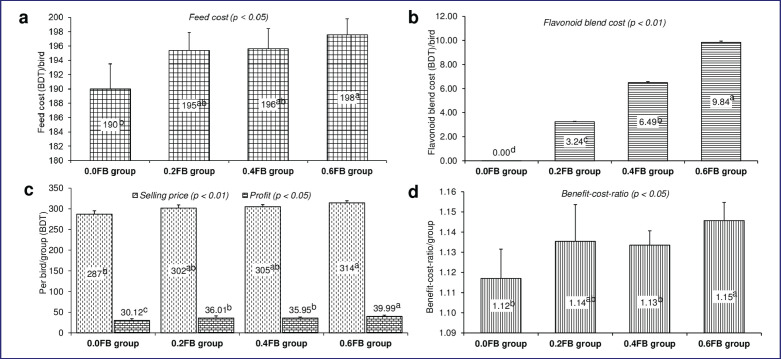
Effect of dietary plant-based flavonoid blend on (a) feed cost, (b) flavonoid blend cost, (c) selling price and profit, (d) benefit-to-cost ratio of broilers (in BDT). ^1^0 FB: basal diet containing mainly corn-soya bean meal without flavonoid blend and having ME = 2982 kcal/kg & CP = 21.61% in starter and ME = 3051 kcal/kg & CP = 20.65% in grower); 0.2 FB: 0.2-gm flavonoid blend/kg diet; 0.4 FB: 0.4-gm flavonoid blend/kg diet; 0.6 FB: 0.6-gm flavonoid blend/kg diet. Other costs were similar among all the groups (*p* = 1.0), i.e., 67 BDT for each bird. 110 BDT = 1 US dollar. ^abcd^means values with dissimilar superscripts differ significantly (*p* < 0.05).

Compared with those in the control and FB groups, birds receiving the 0.6-gm FB per kg diet linearly showed the optimal cost for feed and FB (Fig. 1ab), as well as the maximum bird selling price (Fig. 1c; *p *< 0.01). Moreover, FB supplementation exhibited a linear improvement in both profit (Fig. 1c) and the BCR (Fig. 1d), with the 0.6 FB group presenting the most superior performance in these economic parameters compared with the control (*p* < 0.05).

## Discussion

It is well established that individual flavonoids, when administered at optimal doses, can effectively and economically enhance broiler performance and health. For instance, supplementation with flavonoids, such as 0.5 gm rutin [9], 0.25–1.0-gm quercetin [11], 0.2-gm liquorice [25], or 0.25%–0.75% Arabic gum [14] individually in a per kg broiler diet has been shown to substantially improve growth performance, nutrient digestibility, and gut health. Flavonoids, namely, rutin, promote growth via the IGF-1 receptor pathway [10], while quercetin supports beneficial gut bacteria such as *Lactobacillus* by inhibiting pathogens [11], contributing to improved feed utilization. These benefits align with the present study’s findings, where the FB, containing both flavonoids, resulted in enhanced FI and showed better FCR, CPCR, and MECR. Moreover, individually either rutin or quercetin helps to mitigate dysbiosis by inhibiting the growth of *Staphylococcus aureus*, *Salmonella typhimurium*, and *E. coli*, ultimately enhancing feed efficiency [26]. Likewise, Arabic gum potentially combats gut dysbiosis by promoting probiotics, modulating microbial populations, and boosting short-chain fatty acids (SCFAs) production [27]. Additionally, combined isoquercetin and inulin supplementation have also demonstrated synergistic effects in modulating the gut microbiome [28]. Reportedly, a lower gut pH favors the growth of beneficial bacteria, regenerating intestinal villi, enhancing enzyme activity, and improving nutrient digestion and absorption [29]. Flavonoids, such as rutin, quercetin, liquorice, or Arabic gum, individually create a favorable gut environment by reducing harmful bacteria [30-32]. Quercetin, in particular, enhances gut morphology and immunity by disrupting bacterial membranes and promoting beneficial bacteria growth [11]. These findings suggest that the FB used in this study effectively promoted the proliferation of beneficial bacteria, reduced the risk of dysbiosis, and improved nutrient digestibility. This, in turn, enhances the performance and health of broilers, likely due to the synergistic and combined effects of rutin, quercetin, Arabic gum, and liquorice supplementation in the current study.

**Table 2.** Effect of dietary plant-based flavonoid blend on performance traits of broilers.

Variables	Flavonoid blend (FB)^1^/kg diet					*p*-value^2^			
	0	0.2	0.4	0.6		Overall	*L*	*Q*	*C*
IBW (gm)	43.34 ± 0.62	43.18 ± 0.96	43.67 ± 0.79	44.06 ± 1.00		0.63	0.71	0.26	0.66
FBW (gm)	1784^b^ ± 49	1872^ ab^ ± 46	1892^ab^ ± 31	1946^a^ ± 34		0.02	<0.01	0.53	0.42
BWG (gm)	1740^b^ ± 49	1828^ ab^ ± 47	1849^ab^ ± 31	1902^a^ ± 33		0.02	<0.01	0.53	0.42
ADG (gm)	49.73^b^ ± 1.42	52.24^ab^ ± 1.35	52.36^ab^ ± 0.88	54.84^a^ ± 1.49		0.02	<0.01	0.53	0.42
FI (gm)	3114 ± 64	3203 ± 41	3208 ± 45	3240 ± 37		0.09	0.02	0.39	0.33
FCR	1.78^a^ ± 0.01	1.75^bc^ ± 0.02	1.73^ab^ ± 0.01	1.70^c^ ± 0.01		<0.01	<0.01	0.79	0.71
CPCR	0.37^a^ ± 0.003	0.36^ab^ ± 0.005	0.35^bc^ ± 0.003	0.35^c^ ± 0.002		<0.01	<0.01	0.78	0.70
MECR	5.42^a^ ± 0.04	5.32^ab^ ± 0.07	5.25^bc^ ± 0.04	5.16^c^ ± 0.04		<0.01	<0.01	0.80	0.72

IBW: initial body weight; FBW: final body weight; BWG: body weight gain; ADG: average daily gain; FI: feed intake; FCR: feed conversion ratio; CPCR: crude protein conversion ratio; MECR: metabolizable energy conversion ratio; gm: grams; %: percentage. ^1^0 FB: basal diet containing mainly corn-soya bean meal without flavonoid blend and having ME = 2982 kcal/kg & CP = 21.61% in starter and ME = 3051 kcal/kg & CP = 20.65% in grower); 0.2 FB: 0.2-gm flavonoid blend/kg diet; 0.4 FB: 0.4-gm flavonoid blend/kg diet; 0.6 FB: 0.6-gm flavonoid blend/kg diet. ^2^*L*: linear; *Q*: quadratic; *C*: cubic; values of different variables indicate as mean ± standard deviation;^ abc ^means values with dissimilar superscripts differ significantly (*p *< 0.05).

**Table 3.** Effect of dietary plant-based flavonoid blend on apparent nutrient digestibility and cecal microbial population of broilers.

Variables	Flavonoid blend (FB)^1^/kg diet					*p*-value^2^			
	0	0.2	0.4	0.6		Overall	*L*	*Q*	*C*
Apparent nutrient digestibility (%)									
DM	89.45^c^ ± 0.13	90.01^b^ ± 0.19	90.11^b^ ± 0.10	91.75^a^ ± 0.09		<0.01	<0.01	<0.01	<0.01
CP	65.62^c^ ± 1.28	63.78^d^ ± 0.48	67.07^b^ ± 0.02	73.52^a^ ± 0.28		<0.01	<0.01	<0.01	<0.01
EE	77.15^c^ ± 1.33	79.60^c^ ± 0.52	81.37^b^ ± 0.62	83.25^a^ ± 1.41		<0.01	<0.01	<0.01	<0.01
NFE	87.67^c^ ± 0.46	85.84^d^ ± 0.29	88.55^b^ ± 0.20	90.43^a^ ± 0.39		<0.01	<0.01	<0.01	<0.01
Cecal digesta									
pH	7.36^a^ ± 0.09	7.17^a^ ± 0.28	6.60^b^ ± 0.19	6.49^b^ ± 0.19		<0.01	<0.01	0.76	0.14
Cecal microbial population (log10)									
TVC	10.25^a^ ± 0.11	9.87^a^ ± 0.51	7.78^c^ ± 0.46	8.73^b^ ± 0.43		<0.01	<0.01	0.02	<0.01
TECC	6.99^a^ ± 0.49	6.88^a^ ± 0.60	3.84^b^ ± 0.51	4.54^b^ ± 0.63		<0.01	<0.01	0.24	<0.01
TSC	3.41^a^ ± 0.05	3.18^b^ ± 0.06	2.47^c^ ± 0.04	2.54^c^ ± 0.02		<0.01	<0.01	<0.01	<0.01
TScC	8.05^a^ ± 0.47	6.79^b^ ± 0.34	4.71^c^ ± 0.55	4.58^c^ ± 0.42		<0.01	<0.01	0.06	0.04
*Lact. *spp.	7.09^b^ ± 0.54	9.61^a^ ± 0.54	9.99^a^ ± 0.53	9.66^a^ ± 0.55		<0.01	<0.01	<0.01	0.30

CP: crude protein; EE: ether extract; NFE: nitrogen-free extract; TVC: total viable count; TECC: total *E. coli *count; TSC: total *salmonella* count; TScC: total *staphylococcus* count; *Lact*. spp.: *Lactobacillus* species. ^1^0 FB: basal diet containing mainly corn-soya bean meal without flavonoid blend and having ME = 2982 kcal/kg & CP = 21.61% in the starter and ME = 3051 kcal/kg & CP = 20.65% in grower); 0.2 FB: 0.2-gm flavonoid blend/kg diet; 0.4 FB: 0.4-gm flavonoid blend/kg diet; 0.6 FB: 0.6-gm flavonoid blend/kg diet. ^2^*L*: linear; *Q*: quadratic; *C*: cubic; values of different variables indicate as mean ± standard deviation;^ abcd^mean values with dissimilar superscripts differ significantly (*p *< 0.05).

**Table 4.** Effect of dietary plant-based flavonoid blend on serum biochemical indices of broilers.

Variables	Flavonoid blend (FB)^1^/kg diet					*p*-value^2^			
	0	0.2	0.4	0.6		Overall	*L*	*Q*	*C*
Serum lipid profile (mg/dl)									
TG	54.51^b^ ± 1.79	66.41^a^ ± 9.87	54.67^b^ ± 5.40	39.88^c^ ± 3.28		<0.01	<0.01	<0.01	0.02
TC	116.03^a^ ± 3.87	99.65^ab^ ± 14.89	87.98^b^ ± 3.02	95.44^b^ ± 13.24		0.05	0.02	0.07	0.59
HDL-C	33.31^b^ ± 3.30	43.62^a^ ± 2.97	46.97^a^ ± 2.72	46.77^a^ ± 5.14		<0.01	<0.01	0.03	0.72
LDL-C	71.81^a^ ± 6.18	42.74^b^ ± 13.46	30.08^b^ ± 3.05	41.02^b^ ± 16.32		0.01	<0.01	0.01	0.80
VLDL-C	10.90^b^ ± 0.35	13.28^a^ ± 1.51	10.93^b^ ± 1.08	7.97^c^ ± 0.65		<0.01	<0.01	<0.01	0.15
Serum protein indices (mg/dl)									
TP	2.28 ± 0.24	2.61 ± 0.34	2.51 ± 0.23	3.41 ± 1.11		0.19	0.06	0.43	0.38
Albumin	0.99^b^ ± 0.06	1.21^b^ ± 0.13	1.69^a^ ± 0.30	1.75^a^ ± 0.13		<0.01	<0.01	<0.01	0.02
Globulin	1.29 ± 0.21	1.40 ± 0.38	0.82 ± 0.23	1.56 ± 0.97		0.05	0.11	0.05	0.08
Uric acid	14.52 ± 0.53	14.23 ± 0.47	14.43 ± 0.61	13.70 ± 0.53		0.32	0.14	0.51	0.33
Liver health status (IU/l)									
ALT	16.68^a^ ± 1.50	12.53^b^ ± 2.55	8.82^c^ ± 2.27	6.30^c^ ± 1.20		<0.01	<0.01	0.50	0.88
AST	6.02^a^ ± 0.62	4.89^ab^ ± 1.55	4.56^ab^ ± 1.77	2.48^b^ ± 1.82		0.10	0.02	0.60	0.53
ALP	39.21^a^ ± 2.71	22.68^b^ ± 6.90	15.65^b^ ± 13.75	7.53^b^ ± 7.35		0.01	<0.01	0.42	0.64
Serum mineral profile (mg/dl)									
Ca	7.55 ± 0.55	7.74 ± 1.46	7.26 ± 0.52	7.38 ± 0.88		0.92	0.69	0.69	0.61
P	4.67 ± 0.57	4.70 ± 0.31	4.55 ± 0.48	4.40 ± 0.43		0.85	0.44	0.74	0.88

TG: triglycerides; TC: total cholesterol; HDL-C: high-density lipoprotein cholesterol; LDL-C: low-density lipoprotein cholesterol; VLDL-C: very low-density lipoprotein cholesterol; TP: total protein; ALT: alanine aminotransferase; AST: aspartate aminotransferase; ALP: alkaline phosphatase; Ca: calcium; P: phosphorus; mg/dl: milligram per deciliter; IU/l: international units per liter. ^1^0 FB: basal diet containing mainly corn-soya bean meal without flavonoid blend and having ME = 2982 kcal/kg & CP = 21.61% in the starter and ME = 3051 kcal/kg & CP = 20.65% in grower); 0.2 FB: 0.2-gm flavonoid blend/kg diet; 0.4 FB: 0.4-gm flavonoid blend/kg diet; 0.6 FB: 0.6-gm flavonoid blend/kg diet. ^2^*L*: linear; *Q*: quadratic; *C*: cubic; values of different variables indicate as mean ± standard deviation;^ abc^means values with dissimilar superscripts differ significantly (*p *< 0.05).

In this study, FB supplementation led to remarkable beneficial changes in the cecal microbiota, enhancing the breakdown of fiber into monosaccharides and increasing the production of SCFAs via fermentation [27]. These SCFAs are known to promote the synthesis of bile salts, which in turn up-regulate gene expression involved in cholesterol metabolism, resulting in reduced serum triglycerides, cholesterol, LDL-C, and VLDL-C levels [33]. Align with this, FB groups either 0.4 FB or 0.6 FB reduced triglycerides, cholesterol, and LDL-C while increasing HDL-C levels compared with the control, consistent with previous research [9,33] showing that supplementation of either rutin individually inhibits 3-hydroxy-3-methylglutaryl-coenzyme A reductase, reducing lipid synthesis via peroxisome proliferator-activated receptors-α activation. Similar effects were observed with liqorice and Arabic gum [34], which support the findings of the current study. However, supplementation of quercetin exhibits inconsistent results on lipid metabolism [33,35]. Moreover, these findings suggest that supplementing broilers with a combination of flavonoids, such as rutin, liquorice, quercetin, and Arabic gum combined, may offer similar benefits, such as reduced lipid metabolism and increased HDL-C levels. In this study, FB supplementation led to a linear and quadratic increase in serum albumin, without affecting serum total protein, globulin, or uric acid levels. Similarly, rutin supplementation showed no impact on serum protein indices, partially supporting our findings [9]. Furthermore, improved CP digestibility in the 0.6 FB group may be the prime reason for enhanced serum albumin levels and nitrogen retention, which contributed to the superior growth performance observed in this group [18,24]. This study also showed hepatoprotective effects, with reduced ALT and AST activity, consistent with [14], who reported similar results using liquorice and Arabic gum in rats and broilers. The improved liver health and greater serum albumin concentrations suggest a lower production of free radicals, indicating enhanced overall health status [24]. This may also contribute to greater growth performance, as well as better FCR, CPCR, and MECR in broilers fed either 0.4-gm or 0.6-gm of FB per kg of basal diet.

**Table 5.** Effect of dietary plant-based flavonoid blend on meat quality and bone characteristics of broiler.

Variables	Flavonoid blend (FB)^1^/kg diet					*p*-value^2^			
	0	0.2	0.4	0.6		Overall	*L*	*Q*	*C*
Dressed yield and breast meat composition (%)									
DY	57.78 ± 3.42	58.43 ± 1.90	59.02 ± 1.81	60.59 ± 2.06		0.45	0.13	0.39	0.36
DM	26.50 ± 0.43	26.64 ± 0.43	26.91 ± 0.62	26.63 ± 0.44		0.78	0.63	0.47	0.61
CP	21.81 ± 0.97	21.90 ± 0.69	22.61 ± 0.48	22.58 ± 0.46		0.37	0.12	0.87	0.46
EE	1.95 ± 0.09	1.92 ± 0.14	2.00 ± 0.11	1.90 ± 0.12		0.74	0.81	0.61	0.36
Meat pH and color									
Meat pH	6.09 ± 0.10	6.12 ± 0.13	6.19 ± 0.11	6.29 ± 0.25		0.47	0.10	0.70	0.34
L*	42.21 ± 0.97	41.70 ± 1.43	41.45 ± 1.09	41.75 ± 0.61		0.84	0.56	0.52	0.92
a*	5.48^b^ ± 0.66	6.37^b^ ± 0.51	7.62^ab^ ± 0.90	8.63^a^ ± 1.19		<0.01	<0.01	0.90	0.01
b*	6.83^a^ ± 1.18	4.78^b^ ± 0.47	4.41^b^ ± 0.38	3.63^b^ ± 0.43		<0.01	<0.01	0.15	0.28
SI	6.57^b^ ± 0.78	8.80^ab^ ± 0.98	7.99^b^ ± 0.21	11.0^a^ ± 1.67		<0.01	<0.01	0.53	0.03
Bones characteristics and mineralization									
SL (cm)	6.50 ± 0 .15	6.07 ± 0.49	6.38 ± 0.10	6.55 ± 0.10		0.19	0.51	0.08	0.22
SD (mm)	8.33 ± 1.23	8.14 ± 0.87	8.45 ± 0.36	8.52 ± 0.60		0.49	0.58	0.24	0.44
SW (gm)	11.49 ± 0.94	12.30 ± 2.11	14.65 ± 1.55	15.37 ± 3.55		0.19	0.82	0.04	0.60
DM (%)	40.17 ± 1.47	40.28 ± 2.45	44.33 ± 1.47	44.84 ± 3.66		0.08	0.02	0.89	0.26
Ash (%)	16.77^b^ ± 0.79	16.96^b^ ± 1.42	19.18^a^ ± 1.41	19.6^a^ ± 1.15		0.04	<0.01	0.87	0.26

DY: dressed yield (without skin); DM: dry matter; CP: crude protein; EE: ether extract; L*: lightness; a*: redness; b*; yellowness; SI: saturation index; SL: shank length; SD: shank diameter; SW: shank weight; cm: centimeter; mm: millimeter; gm: grams; %: percentage. ^1^0 FB: basal diet containing mainly corn-soya bean meal without flavonoid blend and having ME = 2982 kcal/kg & CP = 21.61% in the starter and ME = 3051 kcal/kg & CP 20.65% in grower); 0.2 FB: 0.2-gm flavonoid blend/kg diet; 0.4 FB: 0.4-gm flavonoid blend/kg diet; 0.6 FB: 0.6-gm flavonoid blend/kg diet. ^2^*L*: linear; *Q*: quadratic; *C*: cubic; values of different variables indicate as mean ± standard deviation;^ ab^means values with dissimilar superscripts differ significantly (*p *< 0.05).

In the current research, FB supplementation did not significantly impact dressed yield, meat composition, or L* value, which aligns with previous findings [11,36]. However, the effects of flavonoid or flavonoid-rich herb supplementation have shown inconsistent results on dressed yield, meat composition, and L* value in broilers [18,36]. Additionally, increasing the FB supplementation dose numerically improved dressed yield, meat CP content, and pH value, which is likely due to greater body weight gain, higher nitrogen retention, and a darker meat color in the current study [18,37]. Therefore, a higher meat pH is typically linked to a darker meat color [37], and the present study similarly observed a more red and yellow meat color in the FB groups, with higher concentrations showing more pronounced effects. Rutin, a flavonoid recognized for its potent antioxidant properties [38], can prevent lipid oxidation and pH-related deterioration, thereby improving meat color stability [39]. Moreover, this study demonstrated a higher degree of meat redness in the FB groups, attributed to an increased SI. Flavonoids, with their phytoestrogenic properties, promote tibial and femoral bone growth, leading to enhanced bone formation and increased bone mineral density via Smad1/5/8 and Wnt/β-catenin signaling pathways [40]. Quercetin positively affects bone metabolism, with 0.06% supplementation significantly increasing tibia weight and ash content [41]. In line with these findings, the current study observed a progressive increase in ash percentage with FB supplementation, suggesting improved bone mineralization, likely due to the effects of flavonoids, particularly quercetin.

A healthy gut with a beneficial microbial population facilitated the nutritional digestibility of birds fed FB at varying doses, and improved digestibility exerted positive impacts on the overall growth and meat quality of birds. Out of all the doses, 0.6-gm FB per kg basal diet exhibited the best values for maximum variables. Furthermore, supplementation of 0.6-gm FB per kg of basal diet demonstrated higher profitability and a superior BCR compared to the control group.

## Conclusions

The supplementation of a FB at varying doses with a basal diet resulted in a noticeable improvement in the bird’s performance and nutrient digestibility, with the optimal results observed at 0.6-gm FB per kg of basal diet. However, when administered at 0.4 and 0.6 gm per kg of basal diet, this FB decreased cecal pathogenic bacteria and serum bad cholesterol in birds while increasing beneficial cecal microbial populations, serum good cholesterol, and meat redness. In light of these results, the study recommends 0.6 gm of FB per kg of basal diet to optimize broiler performance and promote economic output.

## List of abbreviations

a*, redness; ALT, alanine aminotransferase; ALP, alkaline phosphatase; AST, aspartate aminotransferase; ANOVA, analysis of variance; b*, yellowness; CP, crude protein; CPCR, crude protein conversion ratio; FB, flavonoid blend; FCR, feed conversion ratio; FI, feed intake, gm, grams; h, hours; HDL-C, high-density lipoprotein cholesterol, L*, lightness; LDL-C, low-density lipoprotein cholesterol; kg, kilograms; ME, metabolizable energy; ME; metabolizable energy conversion ratio; SCFAs, short chain fatty acids; SEM, standard error of mean; SI, saturation index; std., standard deviation; VLDL-C, very low-density lipoprotein cholesterol.
